# Mobility of Spanish nurses in a globalized labor market: the impact on health

**DOI:** 10.1186/1472-6963-14-S2-P145

**Published:** 2014-07-07

**Authors:** M Teresa Icart-Isern, Anna M Pulpón-Segura, Carme Icart-Isern

**Affiliations:** 1 School of Nursing, University of Barcelona, Barcelona 08907, Spain; 2 School of Nursing, University of Barcelona, Barcelona 08907, Spain; 3 ABS Sant Josep, Institut Catala de la Salut, Barcelona 08907, Spain

## 

This abstract attempts to provide a framework to discuss the impact of Spanish nurses’ mobility on health systems by changing the composition of the health workforce in both sending (Spain) and receiving countries (other European countries) [1]. These gains and losses may strengthen or weaken the performance of health systems and, while they may seem negligible, produce visible impacts when numbers increase or through continuous mobility over years.

A survey conducted by the Spanish Council of Nursing Colleges show that nurses’ most significant problem is unemployment, where there were about 21,000 unemployed nurses in 2013, this figure has increased by 209% in the last four years, and it is predicted that there will be more than 75,000 unemployed nurses within five years [2]. Currently nearly 7,000 nurses are working in other countries.

In Europe the demand for nursing is rising as a result of an ageing population; hospitalized patients tend to be much sicker than they used to be and need a higher level of care and as the work is tougher, many experienced nurses are taking early retirement.

Spain is training outstanding professionals. It’s a problem that they leave Spain where there is a shortage of nurses, and many hospitals have to cope with minimum staffing levels. Spain has about 5.41 nurses for every 1,000 inhabitants, compared to 7.97 in the rest of the EU [3]. The principal reason to emigrate is human resources cuts in the Spanish healthcare sector, caused by the crisis. This, coupled with deep spending cuts in health, results in nurses working under temporary contracts with little hope of a permanent position in a hospital and is prompting growing numbers of young nurses, whose training has cost the country millions of Euros (the cost of training a nurse is around €120,000 Euros), to leave to work abroad. Thus, Spain faces a scientific as well as economic loss. The forecast for the next few next years are not at all optimistic. The country is failing to capitalize on highly qualified professionals and the full recovery from this situation will need, at least, a decade.
